# Cloud computing and validation of expandable in silico livers

**DOI:** 10.1186/1752-0509-4-168

**Published:** 2010-12-03

**Authors:** Glen EP Ropella, C Anthony Hunt

**Affiliations:** 1 Tempus Dictum, Inc., Portland, OR, 97202, USA; 2 Department of Bioengineering and Therapeutic Sciences, University of California, San Francisco, CA, 94143-0912, USA

## Abstract

**Background:**

In Silico Livers (ISLs) are works in progress. They are used to challenge multilevel, multi-attribute, mechanistic hypotheses about the hepatic disposition of xenobiotics coupled with hepatic responses. To enhance ISL-to-liver mappings, we added discrete time metabolism, biliary elimination, and bolus dosing features to a previously validated ISL and initiated re-validated experiments that required scaling experiments to use more simulated lobules than previously, more than could be achieved using the local cluster technology. Rather than dramatically increasing the size of our local cluster we undertook the re-validation experiments using the Amazon EC2 cloud platform. So doing required demonstrating the efficacy of scaling a simulation to use more cluster nodes and assessing the scientific equivalence of local cluster validation experiments with those executed using the cloud platform.

**Results:**

The local cluster technology was duplicated in the Amazon EC2 cloud platform. Synthetic modeling protocols were followed to identify a successful parameterization. Experiment sample sizes (number of simulated lobules) on both platforms were 49, 70, 84, and 152 (cloud only). Experimental indistinguishability was demonstrated for ISL outflow profiles of diltiazem using both platforms for experiments consisting of 84 or more samples. The process was analogous to demonstration of results equivalency from two different wet-labs.

**Conclusions:**

The results provide additional evidence that disposition simulations using ISLs can cover the behavior space of liver experiments in distinct experimental contexts (there is in silico-to-wet-lab phenotype similarity). The scientific value of experimenting with multiscale biomedical models has been limited to research groups with access to computer clusters. The availability of cloud technology coupled with the evidence of scientific equivalency has lowered the barrier and will greatly facilitate model sharing as well as provide straightforward tools for scaling simulations to encompass greater detail with no extra investment in hardware.

## Background

The scientific value of multilevel, multiscale, computational, biomedical models will be greatly enhanced by making them broadly available and sufficiently manipulable to address a variety of scientific questions at reasonable costs, regardless of the hardware at the researcher's disposal. The availability of cloud technology opens the door to that eventuality. However, such models are analogous to an entire, specialized, wet laboratory. As with wet laboratories, insuring scientific equivalency of duplicate experimental systems in different laboratories is a necessary precondition for placing confidence in the results of experiments arising from those laboratories. A goal of this project was to test the scientific equivalence of experiments conducted using multilevel, multiscale, In Silico Livers (ISLs) executed on a local cluster with those executed in the Amazon EC2 cloud platform. Equivalence demonstrates that validation results obtained on the local cluster still hold in the cloud. Further scientific work on ISLs executing in the cloud can build upon that work. By executing ISLs in a cloud, we gain more computational power to develop more detailed, larger scale, larger scope ISLs, while outsourcing systems administration and hardware maintenance costs.

**Figure 1:**
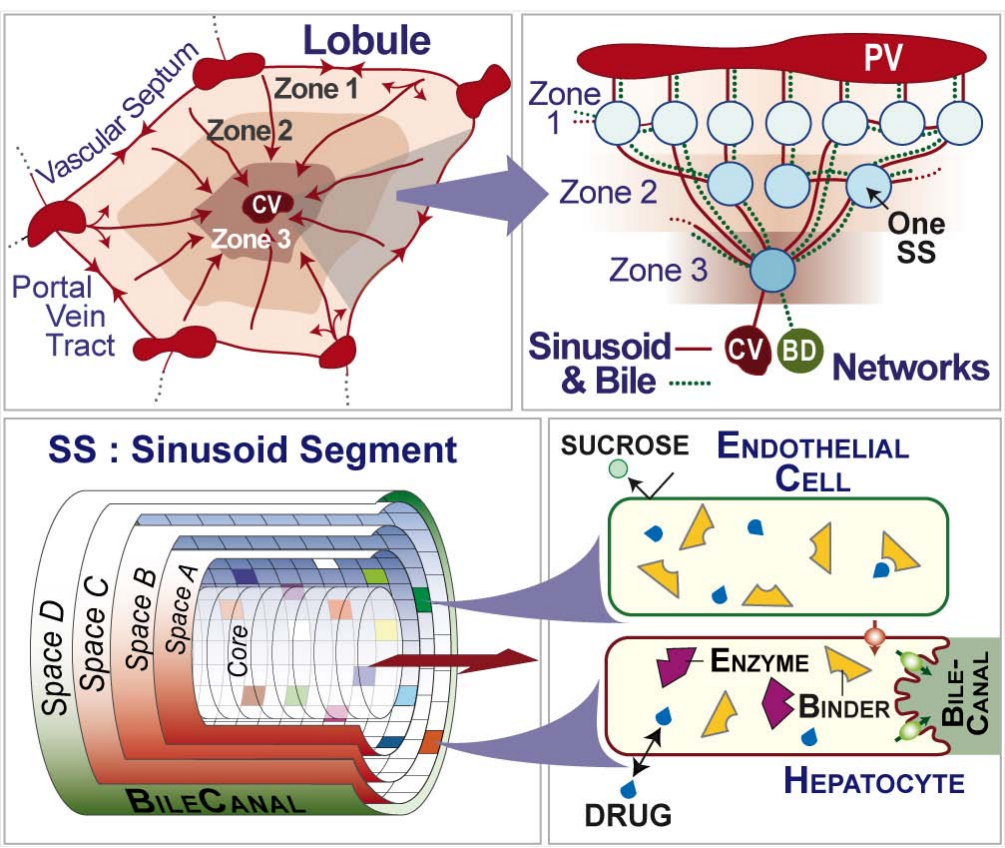
**ISL structure and components**. Descriptions of components and events are provided in the text.

The ISL illustrated in Figure 1 is not intended to be a finalized model having a fixed structure. It is designed to facilitate exploration of plausible mechanistic explanations for observations related to xenobiotic disposition [1-4]. The validation aspect for these experiments is the outflow profile from a single-pass rat liver perfusion experiment [5]. However, concrete instances of multiscale validation by adding finer grained aspects have been reported [6,7]. An ISL is a biomimetic analogue designed to help formulate and challenge mechanistic hypotheses about the hepatic disposition of xenobiotics in health and disease. It is an assembly of abstracted components representing aspects of hepatic form, space, and organization interacting with compounds (a simulated compound; when referring to an ISL feature or component that has a wet-lab counterpart, we use small caps). It has its own unique phenotype, which is necessarily simpler than that of the entire set of lobule mechanisms. Because of the stochastic nature of ISL simulations, each in silico experiment-constructed from several Monte Carlo (MC) lobule samples-generates a slightly different outflow profile.

The modeling and simulation methods used [8,9], described in Methods, are intended for representing large-scale biological systems in silico. They require the computational ability to represent any layer in a hierarchical biological system well enough to falsify (or not) its outputs against corresponding wet-lab observations, and to extend the representation, at will, to any other phenomena necessary to achieve falsification or validation. An ISL cannot be constrained by the use cases or aspects by which it was originally constructed. It must be arbitrarily extensible. It is usually impractical to expect ISLs and similar models to execute in a sequential computing context, e.g., using a single CPU. That is because the depth and scale of most biological systems leads to models that are computationally and analytically insoluble. Inductively derived, equation-based models are more amenable to execution on sequential machines because of their high degree of abstraction from the particulars of the referent mechanisms. At the other end of the computational spectrum, massively parallel machines are also impractical because the solutions that can be implemented are very tightly engineered to match exacting specifications; hence a massively parallel solution is specialized and limited in the extent to which it can propagate [6,10]. In addition, as is evident in Figure 1, the behavior of each lobule is tightly coupled to the behavior of all lobule components. Hence, distribution of the lobule sub-components over multiple, parallel processors would likely result in a very high communication-to-computation ratio, making it inefficient for that type of fine-grained parallelism. In contrast, the salient interactions between lobules, for outflow profile validation data, can be abstracted parsimoniously to lobule input/output: at the portal vein tracts (triads) and the central vein. It is a natural fit to run a single lobule on a single cluster node. For other analogues, we can, for example, envision running single cells on single cluster nodes. Moreover, as demonstrated in [4], highly specific intra-lobule mechanisms can be studied in a very concrete and particular way on a single cluster node. Cluster type machines meet ISL parallelism needs fairly well and have provided the ISL's infrastructure [1-4].

The combination of the liver's homogeneity at a coarse grain and the heterogeneity that is evident at fine grains makes it a good referent system for ISLs built atop cluster parallelism within a cloud. There are three model- and referent-independent reasons for exploring using a cloud infrastructure for cluster style parallelism: 1) reduced systems administration costs, 2) model expansion, and 3) model sharing. ISL uses provide two additional motivations: 4) ISL experiments need many lobule samples, and 5) micromechanistic details need to be assessed.

Although significantly lower cost than biological modeling projects relying on massively parallel machines, those based on local clusters can still present a laboratory with relatively high administrative costs. There are temperature, power, maintenance, and manpower considerations. The resulting constraints limit the extent to which methods suited to cluster computing can be propagated, repeated, and leveraged by others. Local constraints also place practical limits to expanding an analogue's scale and scope.

The advent of publicly accessible, on demand, platform level, cloud computing infrastructures, like Amazon's EC2 raises the accessibility of large scale, expandable, biological models so that anyone with a broadband Internet connection can build virtual clusters and execute such models on demand. They also greatly expand the diversity of in silico, biomimetic devices that can be constructed. The EC2 platform is an interface to an executive web service that allows the on-demand instantiation and management of virtual computers running any of a suite of operating systems that Amazon supports. It is distinguishable from other "cloud" technologies in that platform resources are customizable. As an extreme contrast, Google Documents is also a cloud technology. However, it only allows the user to create particular types of documents and host them remotely on pre-configured servers. At the other extreme, EC2 allows one to instantiate entire virtual machines, running Windows or Linux. There is no limitation on the type of software you can run, unlike with Google Documents. The spectrum of cloud technologies is covered in some depth in [11]. To date, business, collaboration, and data processing dominate the use cases for cloud infrastructures. Although desktop grids have contributed to some scientific domains like molecular dynamics [12,13], recruiting volunteers to allow an application to execute on their computers constrains the researcher in ways that on-demand services do not.

The third and possibly more powerful justification for exploring cloud computing is the potential for sharing models more easily. Often, a simulation is dependent on a particular technology stack that is not widely available or easily configured. With a platform level service like the EC2, virtual machine instances containing the entire technology stack can be shared. When researchers want to examine more than just the published information, they can instantiate a grid of virtual machines, design and execute their own experiments, or simply repeat those of the original laboratory. We can anticipate that, as models and their uses grow and the computational platforms upon which they run scale upward, issues of reproducibility will grow. Methodological details of virtual laboratory assembly and in silico experimental protocols will become as important for computational scientists as they are for scientists experimenting in different wet-labs.

Because each out-flow profile exhibits high variation, it takes averaging many lobule samples to approach the smooth outflow profile exhibited by liver perfusion experiments. A benefit of having an ever-larger number of lobule samples when needed is that the lobular variety within a liver can be approached: an adult rat liver is comprised of a few hundred lobules. Finally, when using the local eight-node cluster (seven slaves and one master), there is a trade-off between abstraction layer for the fine-grained micromechanisms and MC sample size: assessment of the micromechanistic details of the type described in [4] requires larger sample sizes-more lobule samples-to clearly identify event trends.

ISLs are designed for systematic, iterative revision and easy alteration to improve realism and enhance heuristic value. Following revision, the new ISL must be re-parameterized and re-validated using the same criteria used for its predecessor. Addition of new features must not compromise any previously validated behaviors. Planned ISL uses required adding to the most recent ISLs [4] a biliary elimination and flow space, an improved metabolism mechanism, and enabling an alternative method of dose input. Those features were not needed by earlier ISLs. We implemented an ISL that included those features (Figure 1). Observations made during the initial re-validation experiments indicated that we needed to scale experiments to include larger numbers of MC samples. That situation provided an immediate, convenient impetus to move ISL simulations to a Cloud. Results documenting the benefits of such scaling are presented herein. In order to migrate successfully to a cloud, we must demonstrate experimental indistinguishability (defined in Methods) between results from similar size cloud and local cluster experiments. Results of such experiments are presented for the re-validation experiments for ISLs that included the preceding new features.

## Methods

### Middle-out and synthetic modeling and simulation methods

ISLs use middle-out [8] and synthetic [9] modeling and simulation methods. They are specifically intended for representing large-scale biological systems in silico, and are well suited for simulation executions using cloud technology. Middle-out models are a compromise between the long-standing dichotomy between bottom-up and top-down models. Top-down models begin with a high level construct, usually a phenomenon to be generated, and specify its constituents purely from the perspective set by that high level construct. For example, the Navier-Stokes equations for fluid flow are a top-down model because they describe, directly, the aggregate fluid properties of the material being studied, and do not delve down into describing the properties or behavior of the molecules that generate the behavior. Bottom-up models, in contrast, leave the high level aggregate properties of the system unspecified and directly describe the properties of the bottom-most, atomic (devoid of structural information), constituents of the system. For example, a bottom-up model of a fluid would merely describe the molecules and their local interactions without making any attempt to describe the fluid, gas, or solid phenomena at the higher level. Both top-down and bottom-up approaches to modeling implicitly assign an aspect or perspective to the system. That perspective is not necessarily an objective, independently correct perspective. There are layers above any top-down model and layers below any bottom-up model. The modeler chooses where to "pin" the base perspective of the model. Middle-out models accept this a priori perspective and force the modeler to make the model explicit about the aspect chosen as the base. Extensions to the model are then explicitly allowed to go "up" or "down", depending on the requirements set for the uses to which the model will be put. When those layers need to be accessed, it will be easy to do so when using a cloud cluster.

Another spectrum that can be used to describe models and that influences choice of simulation framework is that between inductive and synthetic modeling. Inductive models are derived by abstracting generic properties from many particular situations or objects. Synthetic models, by contrast, are constructed or pieced together from objects available to the modeler. A simple example would be a semblance of an automobile assembled from Lego pieces. These differences are elaborated in [9].

Inductively derived models are more amenable to execution on sequential machines because of their high degree of abstraction from the particulars of the referent mechanisms; but the referent biological systems are typically highly nonlinear and particular. A consequence is that those inductive models are only applicable to more generic contexts and are inapplicable to the specific contexts that will eventually be required to advance pharmaceutical research and support clinical relevance.

ISLs are executed within a co-simulation [3] framework alongside an equation-based, extended, convection dispersion model [14] and an agent that interpolates and outputs the referent wet-lab data. The *ExperAgent *manages all three simulations. Running them in co-simulation allows similarities in behavior to be compared on the fly or after the execution terminates. Comparisons are made via a quantitative Similarity Measure (SM) [1-4]. When ISL observations fail to achieve a prespecified SM, one or more ISL micromechanisms are considered falsified and components or parameters must be modified to generate a new set of micromechanistic hypotheses. A failure to falsify means the mechanisms as implemented represent a plausible hypothesis and we say that ISL has achieved a degree of validation: it validates against the particular aspect quantified by that SM.

### Cluster architecture

**Figure 2:**
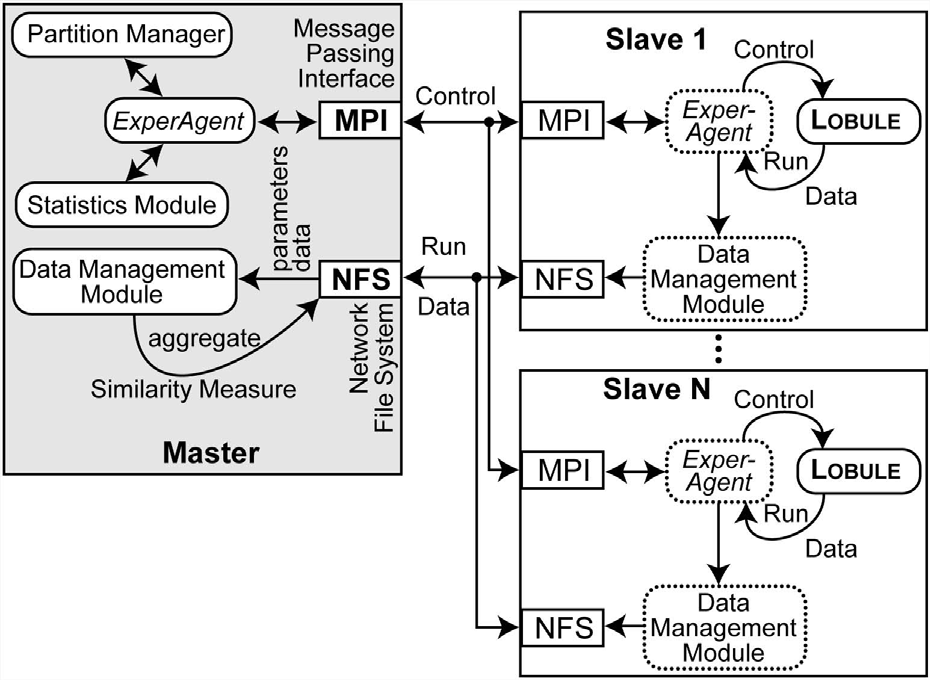
**Components of cluster and cloud architecture**. *ExperAgent *reads parameter data from the Data Management Module and uses the Partition Manager to farm Monte Carlo samples (one per lobule) out to N Slave nodes via the Message Passing Interface. Each Slave node has a proxy copy of the *ExperAgent *and Data Management Module, which writes run data to the Network File System mounted disk drive. Once all samples are finished, the *ExperAgent *sends all the raw observations to the Statistics Module, which sends the derived observations back to the *ExperAgent*. The *ExperAgent *then uses the Data Management Module to write the derived observations to disk.

The architecture is diagrammed in Figure 2. The local cluster consists of eight Intel dual core Pentium 4 based computers, one of which serves as the master node providing the Message Passing Interface (MPI) executive that dispatches jobs to the other seven slave nodes. Each node is connected to a gigabit switch over which it receives commands and data from the master node. The master node contains two network interface cards, one of which provides access to the local area network and Internet, and the other to the Gigabit backplane. The Network File System provides file data and executable to the slave nodes. Execution control and shared memory data are provided via the MPI. The cluster is a Grid in the sense that all the nodes have installed the same operating system, compilers, and libraries.

**Figure 3:**
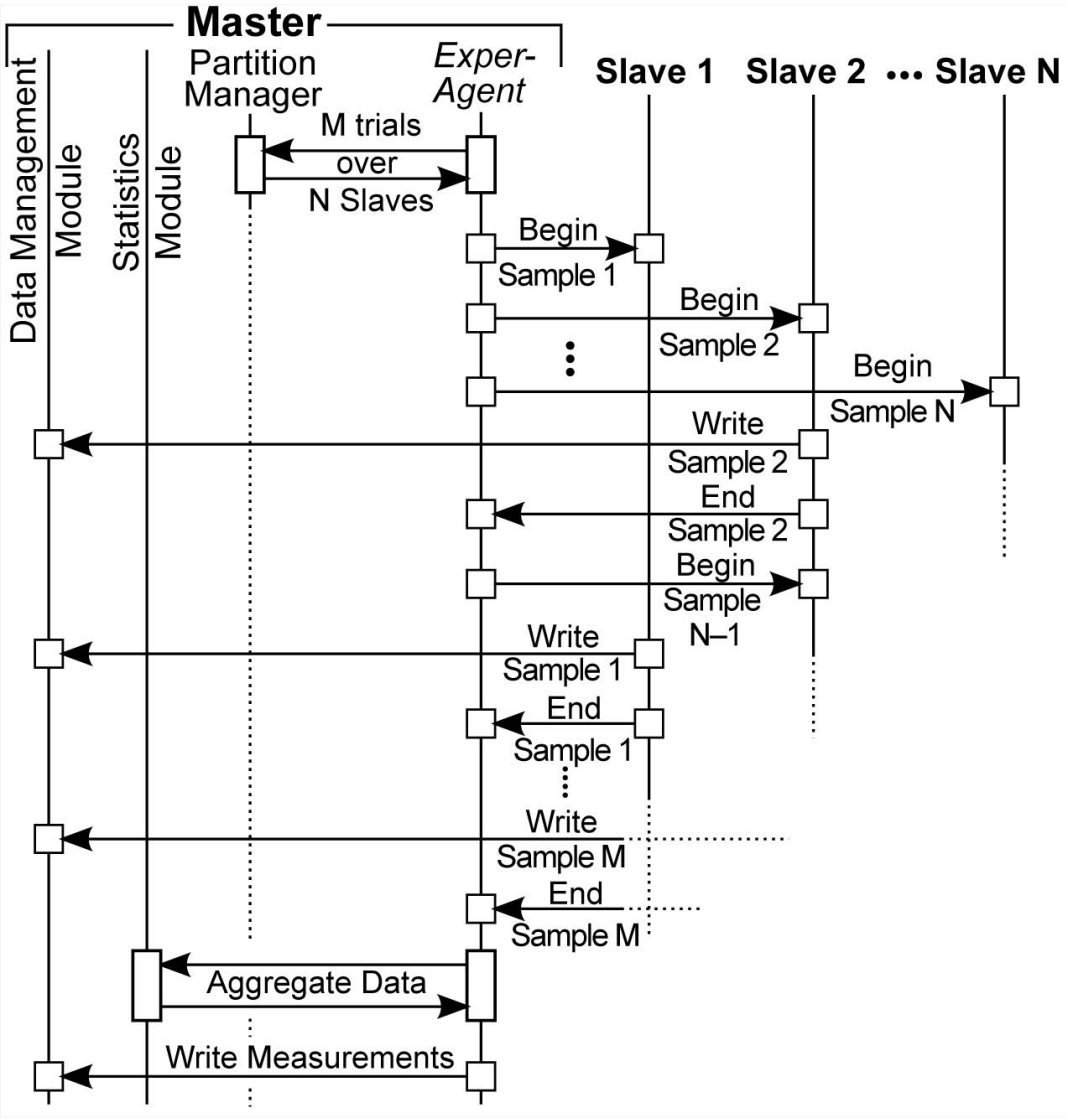
**Event sequence during cluster and cloud execution**. First, *ExperAgent *uses the Partition Manager to calculate which of M samples go to which of N Slave nodes. It then invokes the first sample on the first Slave node and continues until all Slaves are occupied. When any one of the Slaves finishes (asynchronously), it writes the run data for that sample and then signals the *ExperAgent*, which shuts down that sample and spawns a new sample on that Slave. This continues until all M samples are finished, at which point the *ExperAgent *uses the Statistics Module to aggregate the data and calculate the derived observations. *ExperAgent *then writes that data to disk via the Data Management Module.

Each ISL is launched as an executive program on the master node. Event sequences are diagrammed in Figure 3. The executive node instantiates the Experiment Agent (*ExperAgent*). It partitions the simulation among the slave nodes based on command line parameters and the contents of parameter files. For the experiments reported herein, one MC lobule sample is executed per slave node and the results are collected and maintained on the master node until all lobule executions finish. However, the framework also supports a coarser parallelism where whole experiments are executed on a single node. The coarser mode is useful for parameter sweeping and behavior space searching, restricted to experiments with few MC samples.

Only the *ExperAgent *and its utility components execute on the master node. The lobule and all the modeling components having wet-lab experiment counterparts, execute on the slaves. Each lobule sample has a proxy for the *ExperAgent *and the Data Management Module that handle execution control and data I/O. It is important to note that agency is orthogonal to the cluster architecture. The other agents within an ISL (cells and Sinusoidal Segments, SSs) execute serially with interleaved discrete event schedules using the Swarm (http://swarm.org) scheduling engine.

### Similarity Measures and co-simulation

As a lobule executes, the observations taken in compliance with the co-simulation SM are sent to and logged by the *ExperAgent *on the master node. Co-simulation and the SM are described in detail in [1-4]. Briefly, the co-simulation framework requires that any model be executed in tandem with another, a mechanistically different but behaviorally similar model, where the measurement protocol is identical for each co-simulation model. For ISLs, we use two other models, a two compartment ODE model and one interpolated from wet-lab experimental data. The SM provides a ± coefficient of variation band around a nominal profile derived from the wet-lab data. The SM score is the percentage of observations from the simulation that fall within that band. Results of one independent wet-lab experiment are experimentally indistinguishable from another when a prespecified degree of similarity is achieved. Yan et al. [1,2] and Park et al. [3,4] specified SM scores (e.g., ≥ 80%) that must be achieved to establish experimental indistinguishability between results from independent ISL experiments. When a lobule execution finishes, the *ExperAgent *shuts it down and dispatches the next one. When all samples are finished, the *ExperAgent *sends the raw observations to the Statistics Module. It calculates the derived measures that will be used to compare the results for validation or falsification. In the experiments reported herein, the Statistics Module merely averages the results from individual lobule executions and sends that to the Data Management Module to write to the disk for offline analysis.

### Replicating cluster architecture in the cloud

In order to demonstrate experimental indistinguishability between cluster and cloud experiments, the experimental apparatus must be replicated in the cloud. Because an exact replication of the Grid and computation would be degenerate and uninteresting, decisions had to be made about where the cloud architecture would be allowed to differ from the cluster. Amazon's EC2 uses virtual machines running within a farm of actual machines. This means that some or even all the virtual machines, though unlikely, might be executing on the same actual machine. Hence, it is impractical to guarantee the same inter-node network as used on the cluster. Likewise, even though the generic specification of the virtual machines, like amount of memory and compute capacity, are specifiable, as virtual machines, their behavior will be somewhat dependent on the other virtual machines with which they share the real hardware. There are also constraints placed on virtual machine specification by Amazon's infrastructure. For example, a 32-bit virtual machine has a maximum memory of 1.7 gigabytes, whereas the 32-bit machines used in the cluster have 2 gigabytes of memory. As such, the virtual machines differ from the local cluster machines in almost every way at the hardware layer. Thirty-two bit virtual machine images were used in the cloud to limit precision and register size differences that might be introduced. Networking is handled the same as in the local cluster. However, as mentioned above, we cannot be sure that the maximum potential data flow from one virtual node to another is a Gigabit, given the allocation of hardware by the EC2 system. The master node acts as the network address translator, gateway, and firewall to the slave nodes. On both the cluster and the cloud, the simulation executive invokes MPI facilities that query a file on the operating system listing all network reachable nodes available. The script that starts and sets up the slave instances automatically assembles this file. The machines listed could be anywhere on the Internet. Nevertheless, these experiments were only run using virtual instances in the EC2 cloud. Via MPI, the *ExperAgent *is shared between the master and slave nodes, executing only the appropriate code for master or slave. The *ExperAgent *proxy on each slave makes live observations for its lobule until that execution is terminated. When all lobules have terminated, the remaining *ExperAgent *on the master node aggregates the data.

The technology stack for the cloud exactly mimicked that of the cluster. The operating system was Ubuntu 8.04.2, the compiler GNU C/C++/Objective-C 4.2.4, the communications layer MPICH 1.2.7, and the simulation toolkit was the Swarm Subversion trunk from 2009-02-03. Additional file 1 contains instructions for setting up an ISL using EC2. Additional file 2 contains the ISL source code.

The local cluster maintains the state of its hard disk every time it is shut down and restarted. An EC2 machine image, however, does not. The image is a snapshot of a running machine and when virtual machines are instantiated from that image, the state of the hard disk is as it was when the image was created. Hence, although a running instance of an image maintains changes made to the hard disk as long as that instance is running, when it is terminated, all the changes made since it was started are lost. That reality slightly changes the methods for running the ISL in an EC2 cloud.

On the local cluster, if the mechanisms being tested are already in the ISL, then all that is required to run a new experiment is to start the cluster, write the new parameters and/or input data, and execute the simulation. Data written to the disk can be downloaded for analysis immediately, or left on the hard disk for later analysis. The only difference when executing in an EC2 is that the resulting data must be downloaded immediately or it will be lost.

When new or additional mechanisms are to be tested, which was the case for this report, then on the cluster, those changes are made to the ISL source code. Canonical "experiments" are executed as regression tests to verify that the changes work as expected, and the changes are checked into the repository. Thereafter, the experiment proceeds as before. In an EC2 instance, however, because the instance comes up in the state with which it was originally created, it will have no ISL testing capabilities on its disk. If new mechanisms are to be tested, the latest version of the simulation source code must be checked out of the repository to the running virtual machine instance each time a new instance is invoked.

The local cluster could not robustly handle experiments of more than 84 MC lobule samples. So, although the model scales to experiments requiring larger sample sizes, to scale the local cluster, we would have to add more nodes. Because the cloud platform is easy to scale simply by invoking more virtual machines, the 152 sample experiments were possible.

### In Silico Liver structure and components

An ISL during execution maps to a mammalian liver undergoing perfusion as in [14]. An ISL represents a liver as a large, parallel collection of similar lobules, the functional units of a liver. The earlier version, absent BileCanal and the new metabolism mechanism, is detailed in [2,3]. The following is an abridged description. Components mimic essential hepatic form and function features. Flow networks are represented by an interconnected, directed graph, the structure of which is Monte Carlo determined for each lobule. Graph edges specify flow connections between Sinusoidal Segment (SS) placed at each node. There are multiple, different flow paths from portal vein tracts (PV) to CV. Most functions reside within SSs. A SS is a discretized, tube-like structure comprised of a blood "Core" surrounded by three identically sized 2D grids (Spaces A-C), which together simulate a 3D structure. Two SS classes (Table 1) are specified to provide sufficient variety of compound travel paths. Compounds are represented using mobile objects that move through the lobule and interact with encountered SS features. A typical compound maps to many drug molecules. A compound's behavior is determined by the physicochemical properties of its referent compound, along with the lobule and SS features encountered. Multiple, different compounds can percolate through SS features during the same experiment. Objects called cells (two types: endothelial cells and hepatocytes) map to an unspecified number of cells. They function as containers for other objects. Different colored grid locations in Figure 1 illustrate that the features at any location can be unique. Cells contain a stochastic, parameter-controlled number of binders in a well-stirred space. Binders map to transporters, enzymes, lysosomes, and other cellular material that binds or sequesters drug molecules. A binder within an endothelial cell only binds and later releases a compound. Binders within hepatocytes are called enzymes because they can bind compounds and later either release or metabolize it. Adjacent to Space C is the BileCanal (Space D). It is configured similar to the blood "Core." Objects placed in the BileCanal are collected in the bile duct (BD).

**Table 1:** Listed are the ISL parameters and the values used for this work.

Parameter	Value	Description
*StepsPerCycle*	2	# of model steps in 1 simulation cycle

*SSTypeRatio*	0.550	Ratio of short, wide SS to long, narrow SS

*DirSinCirc*	24	Width of short, wide SS

*DirSinLenAlpha*	1.000	Gamma distribution shape parameter

*DirSinLenBeta*	0.085	Gamma distribution scale parameter

*DirSinLenShift*	0.000	Scalar shift parameter

*TortSinCirc*	10	Width of long, narrow SS

*TortSinLenAlpha*	8.000	Gamma distribution shape parameter

*TortSinLenBeta*	0.075	Gamma distribution scale parameter

*TortSinLenShift*	-10.000	Scalar shift parameter

*SinusoidTurbo*	0.250	Ratio of forward to zero bias for compound random walk

*CoreFlowRate*	1	# SS grid points per cycle compound moves in the Core and the bilecanal

*BileCanalCirc*	1	Thickness of the bilecanal in SS grid points

*S2EJumpProb*	0.850	Likelihood a compound will move from Space A to Space B

*E2SJumpProb*	0.150	Likelihood a compound will move from Space B to Space A

*E2DJumpProb*	0.850	Likelihood a compound will move from Space A to Space C

*D2EJumpProb*	0.150	Likelihood a compound will move from Space C to Space B

*ECDensity*	0.900	Fraction of Space B points filled with endothelial cells

*HepDensity*	0.900	Fraction of Space C points filled with hepatocytes

*BindersPerCellMin/Max*	125/125	Uniformly distributed # of binder objects in each cell

*MetabolismProb*	0.200	Likelihood a bound compound will be metabolized

*SoluteBindingProb*	0.750	Likelihood an intracellularcompound will be bound

*SoluteBindingCycles*	15	# of cycles a compound is bound

*SoluteScale*	1.0	Scaling from ISL to wet-lab dose fraction

*MembraneCrossing*	No/Yes	Whether compound crosses cell membranes

*BileRatio*	0%/50%	Percent of compound that goes back into cell vs. bile

*Dosage*	5,000	Generate and insert this many compound objects

*DosageCycle*	2	Simulation cycle at which to inject the compound

### Experiments

Park et al. [3] reported an ISL and parameterization that validated against diltiazem outflow profiles from single pass, perfused, normal rat livers. The same wet-lab data serves as referent for the experiments reported herein. The simulation methods and iterative refinement protocol are also the same. However, for the experiments reported herein, two mechanisms were added and one mechanism modified. The former were discrete time metabolism and the bile canaliculi (BileCanal) illustrated in Figure 1. Previous studies used a discrete event model for metabolism where an enzyme tested a pseudo-random number draw only when *SoluteBindingCycles *had passed. Although this micromechanism cannot be falsified against the available, coarse validation data, it has been pointed out that it is overly discrete and may produce abiotic artifacts. The experiments reported herein used a discrete time model for metabolism such that after binding, an enzyme determines if metabolism has occurred (or not) every simulation cycle up to and including the last before it releases the compound. This micromechanism provides more variation in the event history of the compound and more opportunity for metabolism. Consequently, these change require re-parameterization and re-validation.

Previous studies did not include the creation of a metabolite because having it was not necessary for validation; when a metabolic event occurred, the compound was simply destroyed. However, it has been pointed out that the omission of this micromechanism detracts from the believability of the model. Because believability is closely tied to falsification and validation, the addition of this micromechanism adds heuristic value. The new micromechanism constructs a new metabolite when an enzyme successfully metabolizes a compound. A pseudo-random number draw is tested against the *BileRatio *parameter specific to the compound (diltiazem in this case). The hepatocyte decides whether the metabolite goes into the BileCanal or into the intracellular space. The dynamics of the BileCanal are identical to the Core. Once inside the BileCanal, solute steps forward each simulation cycle the number of spaces designated by *CoreFlowRate*. When a metabolite in the BileCanal reaches the outlet of a SS it moves to the BileCanal inside the next SS. If the outlet goes to the CV, the metabolite is moved, logged, and destroyed. That process maps to metabolite in bile entering the bile duct. The BileCanal is specified with a "circumference" as if it, like Space A, had a two dimensional grid wrapped around it. This geometry is used solely as a constraint to estimate how many metabolites may flow from a predecessor SS to a successor SS. It is not used when metabolite enters the BileCanal or when all metabolites are pushed forward within the data structure. So doing provides a minimal model for the constraints on bile flow and its output.

A manual search of the parameter space for a parameter vector that satisfies the SM produced the parameters in Table 1. Of particular importance are *BileCanalCirc*, *BileRatio*, *SoluteBindingCycles*, *MetabolismProb*, *BindersPerCell*, *DosageParams*, and the various values for *JumpProb*. A value of 1 for *BileCanalCirc *means that only a single compound can flow from a predecessor SS to a successor SS in a single step. Note that *BileRatio *is compound specific and is 0.5 for metabolizable diltiazem but 0.0 for both sucrose and metabolite; because sucrose does not enter cells and enzymes cannot bind metabolites (unless configured to do so), their *BileRatio *values are irrelevant. The *DosageParam *values for these experiments are set so that dosage is an impulse and all compounds are created and injected into the PV in a single simulation cycle. It is also important to note that the *JumpProb *values in Table 1 are biased significantly outward so that the tendency is for compound to move from Space A to Space C.

Manual searches were performed on the cluster using only 28 MC samples, which produced outflow profiles that were smooth enough for decision-making, but were not smooth enough to satisfy the SM. When a 28-sample result showed promise, experiments having 49 or more samples were undertaken to actually test the hypothesis that the parameterized mechanisms will produce outflow profiles that achieve the SM. In the latter experiments, the point was only secondarily to validate against the wet-lab data given the new mechanisms. The primary objective was to test the equivalence of the cluster and EC2 platforms. In order to do so, the parameter vector would have to produce a pair of results that validate in both the cluster and the EC2. If either pair of profiles, from the cluster or cloud, failed to validate, then there was a significant experimental distinction between the local cluster and EC2.

In principle the simulation could be configured to produce identical outputs when run with identical pseudo-random number seeds on both platforms. However, it is important to note that such identical experiments should be explicitly avoided. Determinism is critical for software engineering and verification, where the computational system is not the subject of experimental exploration and hypothesis falsification. As laid out in [9], in order to synthesize heuristically valuable computational analogues that are useful for mechanism discovery and falsification, the system must be suitable for experimentation, just as in vitro systems are the objects of experimentation for wet-lab studies. Further, for simulations where the coupling is tight between the context (or use-case) and the internal mechanisms, as it is when identical deterministic results are expected of a simulation, the model tends to be too abstract, inductive, and very fragile to context [9]. The method of repeating stochastic experiments where some elements are tightly controlled and others are completely uncontrolled is more biomimetic and it mimics wet-lab methods more closely while enhancing scientific impact and usefulness. For these reasons, the experiments reported herein use the full stochasticity of the ISLs over and above the underlying platform differences and place all emphasis on experimental distinguishability or lack thereof.

## Results

### Validation of an enhanced ISL

To demonstrate methodological scientific equivalence, we must use the same model scaling and validation/falsification method on both platforms. To improve realism and heuristic value, we altered the ISL phenotype by making three changes: a biliary elimination and flow space, an improved metabolism mechanism, and an alternative method of dose input. Changes to in vitro models can have unintended consequences. The same is true with multiscale models like ISLs. Because the character of an ISL outflow profile is a consequence of networked micromechanisms, change can alter previously validated behaviors. Thus, new validation evidence is required. The enhanced ISLs that were executed on the local cluster showed a potentially abiotic behavior that had two possible causes. 1) It was a consequence of micromechanisms that were unintentionally altered by the enhancements, or 2) it was an artifact of a small lobule sample size. To test the latter, we needed the option to scale the execution platform to handle a larger number of lobule samples. Rather than buying more hardware to add to the local cluster, we chose to replicate the cluster architecture in the cloud.

### Validation experiments: local and cloud clusters

**Figure 4:**
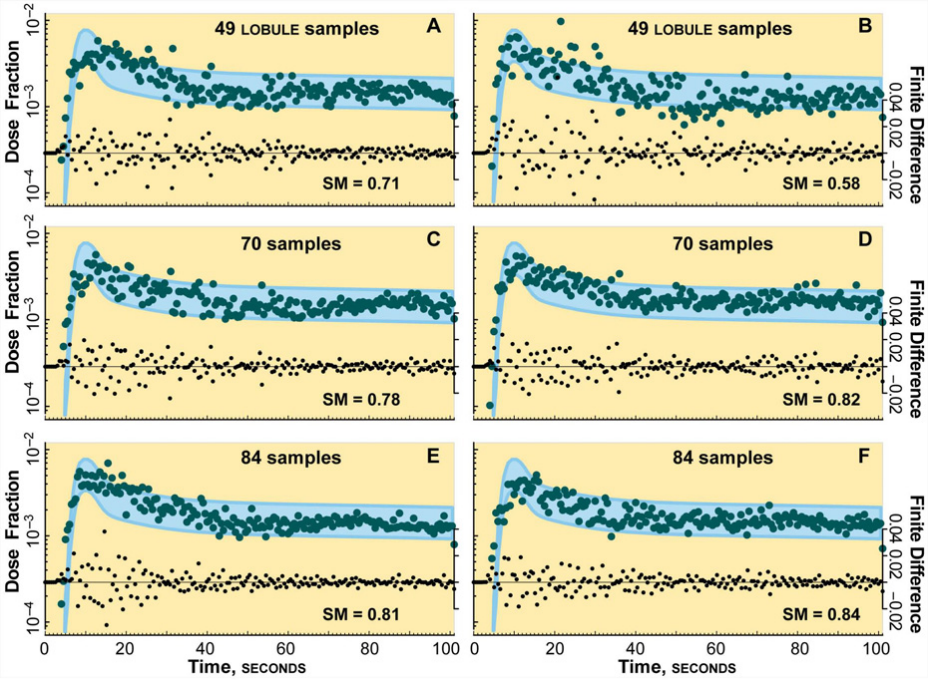
**Cluster execution results**. Shown are results from two ISL experiments on the eight-node, local cluster using 49, 70, 84, and 152 lobules. The fraction of the diltiazem dose collected at CV for each 0.5 s collection interval is plotted. A simulated profile (green circles) is acceptable if SM ≥ 0.8, i.e., ≥ 80% of values fall within the blue zone. An acceptable profile validates that ISL mechanism; an unacceptable profile falsifies it. The center of the zone corresponds to diltiazem blood levels, d [15]. The upper and lower bounds of the target zone are d ± σ/μ (coefficient of variation). σ/μ = 0.334 was calculated as described previously [1]. Smaller black dots are finite differences d_*n *_- d_*n-1*_; they show the extent to which the profile approaches an asymptote, amplifying periodic components. (**A**) shows a long period, exhibited by a local minimum at ~50 and a local maximum at ~80 s. (**B**) shows an oscillation with a local minimum at ~55 and a local maximum at ~95 s, and has the smallest SM value. Increasing lobule samples from 49 to 70 in (**C**) and (**D**) resulted in a less pronounced oscillation, reduced variance, and improved SM values. Increasing sample size to 84 in (**E**) and (**F**) achieved validation, yet there is still evidence of an oscillation, which is more prominent in (**E**). The finite difference profiles are better behaved than in (**C**) and (**D**). We hypothesize that, as the number of samples increases toward the number of lobules in a rat liver, the profile approaches an asymptote smoothly and oscillations disappear. The number of samples was chosen as integer factors of 7 to spread the samples evenly over the 7 slave nodes. 84 samples were the maximum for the local cluster before the memory footprint would cause machine faults.

**Figure 5:**
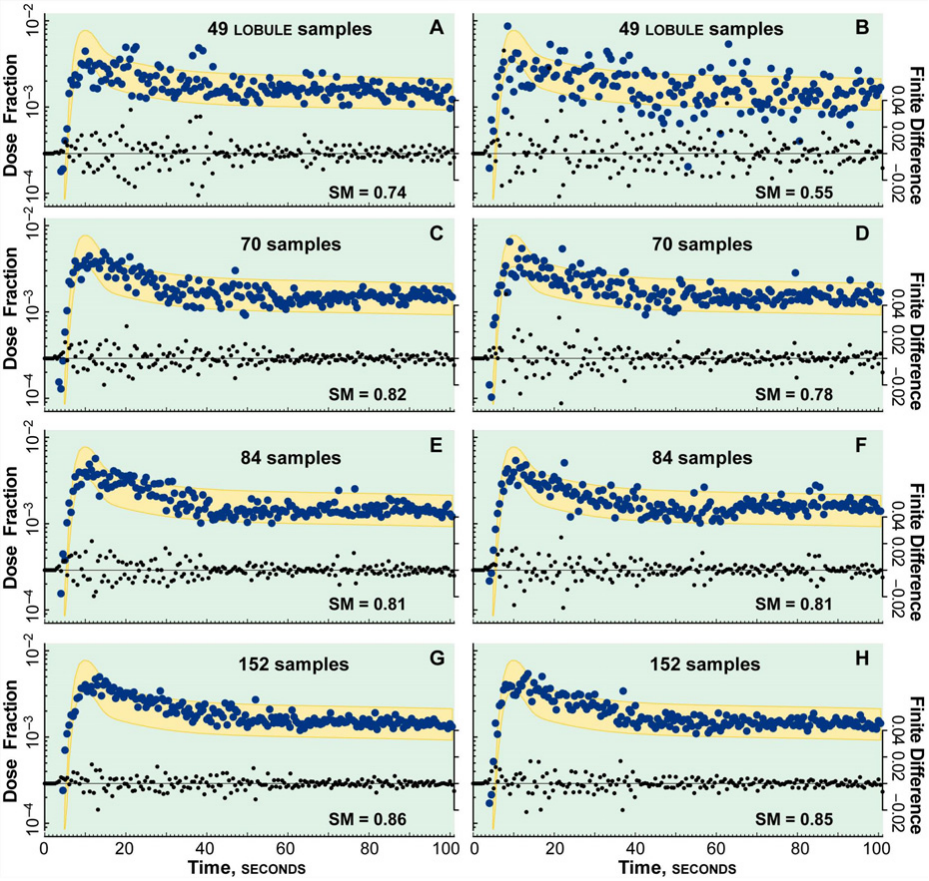
**Cloud execution results**. Shown are results from two ISL experiments using 49, 70, 84, and 152 lobules executed in Amazon's EC2 cloud infrastructure. The values plotted are the same type as in Fig. 4. The gold zone is identical to the blue zone in Fig. 4. As in Fig. 4, in **A**-**D **we see evidence a long period oscillation in the diltiazem outflow profiles. Again, however, the more samples per experiment, the less pronounced the periodicity and the better-behaved the finite differences profiles became: the smoothness of the diltiazem profile increases with the number of samples. More slave nodes could be instantiated to execute more samples per ISL in the cloud. Twenty nodes, one master and 19 slaves, were used to generate the 152-sample ISL simulations.

Experiment results are shown for the local cluster in Figure 4 and for the cloud cluster in Figure 5. The outflow profile for diltiazem is superimposed on the target zone, which designates the SM band derived from wet-lab data, as described above. As with the initial experiments in [3], when SM ≥ 80% the ISL and wet-lab profiles are designated experimentally indistinguishable. We specified that acceptable demonstration platform equivalence would be when SM ≥ 80% for two independent sets of experiments on both cloud and local cluster.

Outflow profiles for diltiazem, d, in normal livers [15] provide the center for the SM band in Figures 2 and 3. The width of the band is derived from the coefficient of variation of the sucrose outflow profiles from [5]. The upper and lower edges of the target zone are {d ± coefficient of variation}. To show the smoothness and asymptoticity of the profiles, the finite differences {d_*n *_- d_*n*__-1_} are also plotted. The outflow profiles for the 49- and 70-sample experiments failed to satisfy the SM in two important ways: the presence of an apparent large period oscillation and high variance. The high variance can be smoothed to show a much more well behaved profile; but for the purposes of this cross validation exercise, the raw data provide more insight into events occurring during the simulation experiments.

The variance in wet-lab outflow profiles is typically rather constant. Deviations about the trend line are typically random, and those observations are the basis for SM target bands in Figures 4 and 5. However, the patterns in Figures 5A, B and 5A, B are different: all four, 49-sample studies showed significant oscillations in variance prior to about 60 seconds that appeared somewhat abiotic. The apparent variance oscillations caused fluctuations in the outflow profiles. One of the runs in cluster and cloud (Figure 4B and Figure 5B) exhibited strikingly large variances. The variance oscillations are obvious in the finite difference scatter plot in Figure 5A. The outflow peak is reinforced by the first peak of the oscillation, making the leading edge of the profile seem markedly false in comparison to the peak shape of the validation band, which is sharper. The apparent oscillation in variance reaches minima at approximately 20, 35, and 50 seconds in Figure 4A; it reaches minima at approximately 30 and 50 seconds in Figure 5A.

Variance oscillations were still evident in the 70-sample local cluster and cloud studies, but overall variance decreased. Because of the ISL's structure, as the number of lobule samples increased, the variance decreased and the asymptoticity increased. That expected trend is evident in the finite difference plots.

Both pairs of 84-sample studies (Figures 4A, B and Figure 5A, B) achieved validation. Although hints of the variance oscillation remain in the 84-sample outflow plots, they satisfied the SM. Further, the variance oscillation is faint or absent in the 152 sample experiments executed in the cloud (Figure 5G, H).

## Discussion

Arguably, the more important methodological impact of validating these ISLs in both the local cluster and EC2 cloud platforms is on model sharing and computational experiment repeatability amongst various laboratories and collaborators. It is a simple matter to acquire and read the source code for a simulation like the ISL. For an organization with ample resources, the appropriate systems administration, and programming skills, it is straightforward to set up a serial computer platform on which to execute an experiment. However, because synthetic models like ISLs are very concrete and implement fine grained, multilevel and multiscale networked mechanisms, experiments on serial computers are infeasible. Similar models will face the same problem. Hence, in order for a laboratory to repeat a given experiment or, more importantly, engage in its own exploration of the analogue, it must have access to a cluster and enough privileged user access to configure the tool-chain on that cluster. This obstacle is higher than it seems, for it requires a well-organized laboratory and the motivation and resources to do the work. In effect, this obstacle contributes to the dominance of engineering oriented modeling and simulation (typically relying on proprietary software), and limits scientific, exploratory modeling to a privileged elite with large budgets.

Although Amazon's EC2 infrastructure costs some money, the semi-automated methods used to instantiate virtual machine images, terminate those instances, and pay for the service with a credit card allow anyone to construct and use their own cluster at minimal cost. During the exploration phase of this work, an individual 49-sample experiment cost approximately $30, representing what a curious scientist might pay simply to instantiate and run a single experiment. When compared with the cost of building, purchasing, or maintaining a cluster, or acquiring time on another laboratory's cluster and then arranging for it to be configured properly for these experiments, the EC2 costs seem quite low. However, this cost estimate is informal. Kondo et al. [11] reported a detailed treatment of costs for the EC2. Costs have also been reported for voluntary computing grid platforms like BOINC [16].

Even though we attempt to replicate as much of the environment as possible, our objective, detailed in Methods, was scientific repeatability, not data duplication. Previous experiments [2-4] only required 49 samples to show experimental indistinguishability, in part because prolonged rather than bolus dosing was used. However, some anomalous results from previous experiments had shown periodicity [17]. Nevertheless, the 49- and 70-sample experiments in both the cluster and cloud failed to validate because of large variance oscillations. We conjectured that perhaps because the diltiazem outflow profile was relatively flat, the ISL parameterizations needed to validate against it would show underlying oscillations that are not evident in profiles that decline more steeply. Something causes ISL outflow profile variance to fluctuate. It could be: a) the parameters, code, or input data differ between each experiment or b) the discretized nature of each lobule is sufficiently unique so that it takes a large lobule sample to produce an acceptable approximation of a continuous liver outflow profile. If it is a), then the apparent artifact would still be evident after a large number of samples; it would not be steadily washed out with additional samples. If it is b), then having experiments comprised of enough samples will make the oscillations cancel each other. The latter is what we observed; we needed at least 84 lobule samples (Figs. 4 and 5) to achieve validation.

One way to conceptualize a discretized ISL during operation is as a large set of oscillators. Each space within an ISL acts, to some extent, like a timed storage device. A compound comes into a space (e.g., Space C), is effectively sequestered there for a number of cycles as it moves about, and it then exits that space. It may or may not be sequestered again later along its path to the CV. Hence each compound goes through a series of store-release processes, except that the store and release events are stochastic. The delay *SoluteBindingCycles*, however, is not stochastic. Each lobule execution starts with 5,000 compounds pulsed into the PV. Each one encounters a large array of "inductors" that can retain a compound for an interval and then release it. When a large number of compounds get caught, released, caught, and released all at roughly the same intervals, we see what looks like a stochastic oscillator with a random signal riding atop a larger oscillation. Such a signal is made more obvious by the impulse bolus and a small number of compounds. The only elements of the model interfering with the predictability of the combined oscillation are: 1) the stochasticity surrounding when each compound is bound and rebound and 2) that some compounds are metabolized and their product moved into the BileCanal, and no longer contribute to the profile. Hence, as the number of compounds increases, the variance oscillations should disappear. Indeed, the high variance that showed up in the 49-sample cluster run in Figure 4B, disappeared when the number of samples were increased to 70. Furthermore, the variance swings diminished in each of the 70 then 84-sample studies on both the local cluster and in the cloud. Finally, for the 152-sample studies in the cloud, the variance swings have disappeared completely and the outflow profile decays more smoothly. The similarity between the cluster and cloud results for 84 and fewer MC lobules demonstrates the equivalence of the models executing on the two platforms. Further, confirming the low sample size conjecture with the 152-sample experiments demonstrates the value of having available the scalable cloud platform to extend the mechanism validation and falsification methods beyond the capabilities of the local cluster.

The results demonstrate experimental indistinguishability of ISL outflow profiles from the local cluster and Amazon EC2 cloud platforms for experiments consisting of 84 or more lobule samples. The results also demonstrated that mechanistic changes confined ISL behaviors to a smaller, more constrained region of parameter space. The modifications provide an even more concrete and specific, falsifiable hypothesis about the referent mechanisms. Making these changes and validating them against the coarse aspect of outflow profiles have made more specific the regions of the parameter and behavior spaces that are biomimetic without losing the generality of ISL parameterization.

## Conclusion

The results contribute evidence of silico-to-wet-lab phenotype similarity, specifically that ISL behaviors can cover some of the behavior space of referent liver experiments. So doing allows for concrete representations of multilevel hepatic mechanisms in many distinct experimental contexts, without degrading the ability to parameterize an ISL so as to accurately represent a particular context. To date, the scientific benefits of experimenting with multiscale biomedical models has been limited to small numbers of researchers with access to computer clusters. Cloud technology coupled with the evidence of scientific equivalency has lowered the barrier and will greatly facilitate model sharing while providing straightforward tools for scaling simulations to encompass greater detail with no extra investment in hardware. The flexibility and dynamic expandability of a platform cloud infrastructure is important for open-ended, exploratory, mechanism-focused research.

## Abbreviations

CV: central vein; ISL: In Silico Liver; MC: Monte Carlo; MPI: Message Passing Interface; M&S: modeling and simulation; PV: portal vein tracts; SM: similarity measure; SS: Sinusoidal Segment

## Authors' contributions

GEPR replicated the cluster architecture in the cloud. GEPR designed and conducted validation experiments. GEPR and CAH evaluated validation experiments. GEPR and CAH wrote, read, and approved the final manuscript.

## Supplementary Material

Additional file 1 **ISL Cloud Supplement**. Instructions for Executing an In Silico Liver (ISL) on EC2. Provided are detailed, step-by-step instructions for setting up an ISL using EC2. Click here for file

Additional file 2 **isl-for-bmc**. ISL Source Code and enabling documents. Click here for file
