# Supplementary material for: Cloud computing and validation of expandable in silico livers
Source: BMC Syst Biol. 2010 Dec 3;4:168. doi: 10.1186/1752-0509-4-168 (PMC3016276; doi:10.1186/1752-0509-4-168)
Supplement: Additional file 1 — ISL Cloud Supplement. Instructions for Executing an In Silico Liver (ISL) on EC2. Provided are detailed, step-by-step instructions for setting up an ISL using EC2. [file 1752-0509-4-168-S1.PDF]

Electronic Supplementary Information
for
Cloud Computing and Validation of Expandable In Silico Livers

Glen E.P. Ropella and C Anthony Hunt

Instructions for Executing an In Silico Liver (ISL) on EC2

1 Start with EC2 standard Ubuntu image
  1.1 Set up an EC2 account at http://aws.amazon.com
  1.2 Choose a Linux image to start with. Any Linux should work; but we used Ubuntu and recommend Ubuntu or Debian.
    1.2.1 We used 8.04 Hardy; but since you're installing from scratch, you may as well try Lucid (10.04) or Maverick (10.10).
  1.3 Start that image
2 Install Swarm on Ubuntu
  2.1 Explicit instructions are at http://www.swarm.org
  2.2 Several standard packages will be required, e.g. automake and gperf. A list of packages we use for Ubuntu 8.04 (Hardy) appear in the file 8.04-packages.txt. Since we compile on 8.10 (Intrepid), we also include the packages we use for 8.10: 8.10-packages.txt. Not all of these packages are required.
  2.3 Download the version of Swarm you want to use. To use the same code we used in this article, the following command should work:

```
svn co -r {2009-02-03} svn://svn.savannah.nongnu.org/swarm/trunk
```

    2.3.1 The install process will differ if you check it out of the repository versus downloading a packaged source or binary distribution, particularly in that the repository version will require you to run the autogen.sh script in the top level directory of the Swarm sandbox.
  2.4 Download one or more of the Objective-C simulation examples, compile it against the new Swarm install, and run it to test that you have the base installed. Note that you may want to use one with a “batch mode” to avoid transmitting GUI data back and forth to the server.
3 Unpack the ISL source code and startup/shutdown scripts with a command like:

```
unzip isl.zip
```

4 Compile the sequential ISL against Swarm
  4.1 Sequential – It simplifies the test of the install if you compile the ISL to run sequentially first.
    4.1.1 Compile - Simply cd into ./isl/src and type make.
    4.1.2 Execute
      4.1.2.1 Run a test sim using the liver1.scm file. That file contains the majority of the parameters for executing an ISL.

```
./liver -b -s -F inputs/parameters/liver1.scm
```

4.1.2.2 Note that you can see what the options mean with the command ./liver –help.

4.2 Parallel

4.2.1 You should be able to compile the parallel ISL as long as you have an implementation of MPI installed, e.g. mpich.

```
make parallel=yes
```

4.2.2 However, to actually run the compiled pISL, you will have to satisfy:

4.2.2.1 actually have multiple machine instances running

4.2.2.2 have the MPI tools installed, especially mpirun.

4.2.2.3 have the slaves listed in the /etc/mpich/machines.LINUX file, and

4.2.2.4 have the slaves and master mount a common NFS partition containing the ISL directory. Edit the ./startnodes.rb script to reflect the NFS partitions you've chosen.

5 Snap master and slave images

5.1 Master: Once you can compile the parallel ISL, create an EC2 image from that running instance. Make sure you can conveniently ssh into the master, preferably with a PKA key pair in a user's ~/.ssh subdirectory. This will make navigation amongst the master and slave nodes transparent.

5.2 Slaves: The slave nodes need not have the same packages installed as the master node. For example, if you compile only on the master, you don't need all the compilation tools on the slaves. However, it is simpler to use the same setup for the slaves as the master. However, for the startup and shutdown scripts included in this supplementary material, you will need to have separate images for the master and slave. You can simply create another image identical to the master for this purpose.

5.3 After you have the master and slave image names, edit the ./cloud-scripts/cloud-lib.rb script with the proper values for the Master_Image and Slave_Image variables.

6 Instantiate the images

6.1 Use the EC2 command line tools or AWS control panel to start the master and ssh into it.

6.2 Ensure that the /etc/mpich/machines.LINUX file is empty because the startup script will add the instance names to the file.

6.3 Use the startnodes.rb Ruby script to start however many nodes you need with a command like:

```
./startnodes.rb 2
```

This will start 2 slave nodes. The number of slaves is a function of either how many Monte-Carlo samples or how many whole experiments you want to run. For example, if you use only a single parameter file (in the isl/src/inputs/parameters/ subdirectory) and the parameter #:monteCarloRuns is 2, then you will be running 2 samples and you can run one on each slave. Alternatively, if you have 2 parameter files, each with #:monteCarloRuns set to 50, then you can execute the experiment for each parameter file on a separate slave node, each of which consists of 50 trials. By default, as of the time of this writing, Amazon will let you run a total of 20 instances, which means you can start 19 slave nodes.

6.4 To ensure that your nodes have started successfully, ssh into each of them and perform some minimal check like:

```
for i in `cat /etc/mpich/machines.LINUX`
do
        ssh $i mount -l -t nfs
done
```

7 Execute

7.1 Parameter files

7.1.1 ./isl/src/inputs/parameters/liver1.scm – This is the default parameter file for the current version of the simulation. liver2.scm is another example, which is not guaranteed to be compatible with this version of the code. For the first attempt to execute an ISL, you should delete the other files in the directory just to be safe. cvsc.scm contains the parameters used for the cloud vs. cluster experiments discussed in the paper.

7.1.2 ./isl/src/inputs/jpet301.ls -- This is the default lobule specification. It specifies the number of nodes per zone and the number of edges between zones. Which lobule spec. file is used is set as the #:artGraphSpecFile parameter in the liver1.scm file.

7.2 Command

7.2.1 The basic command for the parallel ISL is:

```
mpirun -nolocal -np <number_of_slaves> ./liver -b -s
```

This will run each Monte-Carlo sample on a separate machine listed in the /etc/mpich/machines.LINUX file. To find out what the options for the mpirun command mean, use man mpirun. Briefly, the -nolocal option tells MPI not to run a MC sample on the master node. The np <number_of_slaves> option tells MPI to use the first <number_of_slaves> instances from the /etc/mpich/machines.LINUX file in the computation.

7.2.2 Because remote logins can become frozen or disconnected depending on network behavior, it is wise to use nohup for executing long experiments, e.g.:

```
nohup mpirun -nolocal -np 2 ./liver -b -s 2>&1 &
```

This will cause the standard output to go to a file called nohup.out and make the process resistant to disconnects of the login shell. The "2>&1" term tells the shell to reroute standard error output to the same place as the standard output, "nohup.out".

7.2.3 To monitor the execution, follow the above command with:

```
tail -f nohup.out
```

If the login disconnects, simply ssh back into the master node and tail -f nohup.out again to resume.

8 Data Files – When the analog finishes executing, the following directories and files will be available:

Directory	File	Description
isl/src/debugs/	debug-liver1.scm	Debugging information
sl/src/inputs/	jpet301-fig1.csv	Validation data input
	jpet301.ls	Lobule spec. parameters
isl/src/inputs/parameters/	liver1.scm	Main parameters
isl/src/monitors/	monitor-liver1.scm	Execution monitoring for verification
isl/src/outputs/	amount_0000_0000.csv	Solute amount time series' for sample 1
	amount_0000_0001.csv	Solute amount time series' for sample 2
	amount_metab_0000_000?.csv	Metabolized compound for samples 1&2
	art_results.csv	Articulated/Experimental model output
	dat_results.csv	Validation data model output
	dose_0000_000?.csv	Record of dosage
	enzymes_0000_000?.csv	Record of enzyme counts
	graph_0_?.csv	SS nodes per zone table
	graph_0_?.gml	GML description of the SS network
	jpet301_0.ls	Lobule spec. output for verification
	pm0000.scm	Main parameters output for verification
	ref_results.csv	Reference model output
	run_0000_000?.csv	Record of model outputs per iteration
	similarity_results.csv	Similarity Measure (SM) scores
	similarity_series.csv	Ephemera used for SM

Table 1: Output file names and descriptions. There are some other files present that are not listed because they are unused.

9 Shut down the nodes

9.1 Shut down the slaves from the master with the command:

```
./stopnodes.pl
```

9.2 Shut down the master node either with the EC2 scripts on your local machine or via the AWS control panel.
